# University students’ self-assessment of data literacy: A validation study

**DOI:** 10.1371/journal.pone.0322104

**Published:** 2025-04-28

**Authors:** Jeonghyun Kim, Lingzi Hong, Ayoung Yoon

**Affiliations:** 1 Department of Information Science, University of North Texas, Denton, TX, United States of America; 2 Anuradha and Vikas Sinha Department of Data Science, University of North Texas, Denton, TX, United States of America; 3 Department of Library and Information Science, Indiana University Indianapolis (IUI), Indianapolis, IN, United States of America; National University of Sciences and Technology, PAKISTAN

## Abstract

As data literacy has emerged as a critical skill for professionals across industries, educators in higher education have incorporated it into their curricula and instruction. Understanding and evaluating the factors that shape an individual’s data literacy is important for benchmarking proficiency and tailoring curricula, yet the underlying components and structure of data literacy for students in four-year institutions are unknown. This study validated the Data Literacy Self-Efficacy Scale (DLSES) with 1,816 students enrolled in two four-year institutions. Exploratory and confirmatory factor analyses were conducted to determine the construct of the scale, and the item analysis was used to address the validity of the items on the scale. The exploratory factor analysis identified eight distinct factors comprising 29 items. The results of confirmatory factor analysis showed a good model fit, CFI = 0.994, TLI = 0.994, RMSEA = 0.053, SRMR = 0.044. This study demonstrated the 29-item refined version of the DLSES to be a reliable and valid tool for measuring individuals’ self-efficacy levels for data literacy. Furthermore, the scale could form the basis for curriculum development and help educators design targeted interventions that address specific learning needs.

## Introduction

Data is now part of our everyday lives. As we contend with data’s impact on the nature of communication and knowledge, data literacy has emerged as an essential skill everyone requires in their personal and professional lives. Data literacy is a dynamic and evolving form of literacy and highly socially situated concept [1]. Literacy itself, indeed, is understood as “a collection of communicative and sociocultural practices shared among communities,” [2] and it continues to develop along with societal and technological shifts. This evolution has introduced terms like “information literacy,” “media literacy,” “computer literacy,” and “digital literacy” into our vocabulary, reflecting new demands and advancements. As a natural extension of these concepts, data literacy has recently gained significant attention across various fields, including education, information science, public health, and others [3–5].

In an era witnessing unprecedented data growth, developing data literacy has become critical in higher education, where teaching and research increasingly rely on data-intensive practices. Higher education, especially in four-year institutions, faces growing pressure to prioritize data literacy because of its benefits in a competitive labor market. As workplaces become more data-oriented and AI-driven, data literacy is expected to be one of the most in-demand skills [6,7]. Preparing students with this competency is vital for their workforce transition. Many institutions have taken steps to integrate data literacy into their curricula to address this need. These efforts include establishing dedicated data science departments, introducing data-related majors, offering undergraduate elective courses on data literacy, and expanding library programs to teach data literacy alongside traditional information literacy [8–10]. Such initiative aims to equip students with the skills they need to thrive in a data-driven world.

Given the recognized importance of data literacy, studies have explored the concept and components of data literacy, which is critical for tailoring curricula and training. Data literacy is defined as a multifaceted competency [11–13], combining cognitive abilities, critical thinking, and technical skills. It includes skills in identifying, cleaning, analyzing, and interpreting data; evaluating data sources and reliability; identifying data misuse; and utilizing data tools and technologies. Nevertheless, there is a dearth of systematic empirical work operationalizing this concept and developing reliable methods or scales for its measurement.

Recent research has designed data literacy scales. One example is a Data Literacy Self-Efficacy Scale (DLSES) developed by our research team, designed to measure students’ perceptions of their data literacy competency [14]. This scale was originally designed for use in higher education, but it was only validated in a community college setting. Previous validation efforts emphasized the need for further validation to strengthen the scale’s reliability and assess its applicability in diverse contexts [14,15]. Notably, it was recommended that additional validation should target four-year universities, which provide students with a comprehensive and specialized education, preparing them for professional careers or advanced academic studies.

Therefore, the purpose of the current study is to validate the DLSES with students at four-year institutions, including undergraduate and graduate students, who typically face higher expectations and demands for data literacy competency. The study aims to identify the scale’s underlying factor structure and explore its the validity for a broader application within higher education.

## Materials and methods

### Adapting the DLSES

The original version of the DLSES was created to measure students’ perceived proficiency levels in data literacy. The scale comprised 33 items grouped into ten domains: *Data Awareness*, *Data Collection*, *Data Cleaning*, *Data Analysis*, *Data Visualization*, *Data Storytelling*, *Data Quality Evaluation*, *Data Organization*, *Data Storage*, and *Data Ethics* [14]. In the current study, we included all 33 items from the original scale rather than the 31 items retained in our previous validation; this decision was made because the focus of our current study is not investigating the replicability of the factor structure. Instead, it aims to propose an alternative factor structure for a different population – i.e., students at four-year institutions.

A minor modification was made to the response format. Originally, scale items were rated from novice to expert, but the subjective nature of terms like “novice” and “expert” could bias respondents’ perceptions. To mitigate this, the response categories were revised using the framework proposed by Sherer’s Self-Efficacy Scale [16], allowing participants to respond to the items using a 5-point Likert scale, ranging from “strongly disagree” to “strongly agree.” Each item was phrased with “I believe I can successfully…” to reflect this approach.

The survey questionnaire incorporated this modification along with questions about students’ demographic and academic characteristics, including age, gender, race, year in college, field of study, and enrollment status. It was piloted with a small convenience sample of students to estimate completion time, assess the item’s readability, and evaluate the scale’s usability.

### Procedure

The survey questionnaire, deployed via Qualtrics, was disseminated to students at two institutions classified as “Doctoral Universities: Very High Research Activity” by Carnegie Classification. These institutions are also categorized as “Four-year, Large” with fall enrollment data showing at least 10,000 degree-seeking students in full-time equivalent (FTE) enrollment. As state universities, they offer a greater variety of undergraduate, graduate, and doctoral programs across various fields of study to serve diverse student populations. From February 1, 2024 to March 17, 2024, the invitation to the survey was distributed through college/departmental listservs and a student Facebook group. A total of 2,671 responses were collected. This sample size was deemed sufficient for this study, as it is generally recommended to have at least ten participants for each item in the scale [17].

This study was approved as exempt by the University of North Texas Institutional Review Board and Indiana University Human Research Protection Program.

### Data analysis

Data screening was conducted to make sure that data accurately represented what was measured and all the data met the underlying statistical assumptions. First, careless respondents who either selected the same answer for every item or completed the survey in a very short time were removed [18]. Then, outliers were assessed using univariate z-scores ±3.29 [19] and Mahalanobis distance [17].

After the data cleaning process, a total of 1,816 valid responses remained. This data was then randomly and evenly split for the exploratory factor analysis (EFA) and confirmatory factor analysis (CFA) [20,21]. The latent constructs of the scale were assessed using factor analysis, a critical step for scale validation [22]. Specifically, EFA with the first data half and then replicating the factor structure using CFA. To determine the sampling adequacy of data for factor analysis, the Bartlett’s test of sphericity was conducted [23]. Given the Likert nature of the items, a polychoric correlation matrix was used as an input for EFA. Principal axis factoring and oblique rotation were applied as they do not require the data to be normally distributed [24]. The optional number of factors to retain was determined by using parallel analysis, Very Simple Structure [25], and Velicer Minimum Average Partial (MAP) [26] along with Bayesian Information Criterion (BIC) value [27]. Item deletion and retention were determined based on specific criteria, including factor loadings (with a cutoff of ≥ 0.40), the absence of cross-loading between items, and a minimum of three items for each factor [21,23]. After determining the number of factors and items to retain, each factor was labeled based on the concepts represented by the items in that category. The three authors reached a consensus on the labeling of the factors.

Then CFA was conducted to confirm the factor structure obtained from the EFA. The CFA was implemented with the remaining 908 survey responses using the lavaan package in R (version 4.3.0). The diagonally weighted least squares estimator was implemented to account for the ordered nature of the Likert items. To assess the model’s fit with the data, the following indices and criteria were used: a relative chi-square with a non-significant result (*p* > 0.05), Comparative Fit Index (CFI) > 0.90, Tucker-Lewis Index (TLI) > 0.90, Root Mean Square Error of Approximation (RMSEA) within 0.05 and 0.08, and Standardized Root Mean Square Residual (SRMR) < 0.08 [17,28].

Cronbach’s alpha was employed to assess the consistency of the scale. The minimum acceptable value for Cronbach’s alpha coefficient is 0.70 [21]. Additionally, item-level analysis was conducted to further investigate the performance of individual items within the scale and to identify any potential revisions needed for future validation. For each item, item-level statistics were conducted, including mean, standard deviation, and response distributions. Item-total correlations were calculated to assess how well each item relates to the rest of the scale and its ability to differentiate between respondents with higher or lower overall responses.

## Results

### Participants characteristics

As presented in Table 1, the participants exhibited diverse demographic and educational backgrounds, with almost half falling within the 20–24 age range. This aligns with the fact that nearly 40% of survey participants are undergraduate students. The gender distribution was slightly skewed toward females, with 57.88% female and 39.05% male. The race/ethnicity distribution was also imbalanced, with 42.49% of survey participants being Asian, 38.89% being white, and the remainder comprising other groups.

**Table 1:** Characteristics of survey participants.

	Items	*n*	%
*Demographic characteristics*			
*Age*	18–19	210	12.02%
	20–21	290	16.60%
	22–24	568	32.51%
	25–29	384	21.98%
	30–39	178	10.19%
	40–49	84	4.81%
	>50	33	1.89%
*Gender*	Female	999	57.88%
	Male	674	39.05%
	Non-binary	53	3.07%
*Race*	White	660	38.89%
	Hispanic/Latino	135	7.96%
	Black or African American	119	7.01%
	Asian	721	42.49%
	American Indian or Alaska Native	17	1.00%
	Pacific Islander	1	0.06%
	Multiple	44	2.59%
*Educational characteristics*			
*Enrollment status*	Full-time	1401	80.42%
	Less than full-time	341	19.58%
*Academic terms enrolled*	Undergraduate 1st year	128	7.35%
	Undergraduate 2nd year	180	10.33%
	Undergraduate 3rd year	189	10.85%
	Undergraduate 4th year	177	10.16%
	Graduate master	901	51.72%
	Graduate doctoral	147	8.44%
	Non-degree & Other	20	1.15%
*Field of study*	Education	48	2.80%
	Engineering	34	1.98%
	Humanities and Arts	85	4.96%
	Psychology and Social Sciences	53	3.09%
	Other science/engineering and unknown	399	23.29%
	Science	1043	60.89%
	Technologies	51	2.98%

Students from various academic majors, such as computer science, business analytics, accounting, education, psychology, history, criminal justice, and economics participated in the survey. After converting the primary academic major input into the Integrated Postsecondary Education Data System (IPEDS) code, it was found that 60.89% were from science majors, encompassing fields such as computer science, health science, and life science, followed by other non-science and engineering majors. Among these students, 80.42% were full-time, and 19.58% were part-time.

### Exploratory factor analysis results

The KMO was 0.95, well above the commonly accepted threshold of 0.6 [19], indicating that the sample size is sufficient. Bartlett’s test chi-square was statistically significant, with χ² (528) = 39784.978, *p* < 0.001, suggesting that factor analysis was appropriate [19].

The EFA yielded a 29-item instrument that accounted for 76% of the variance in the scale. Four of the original 33 items were dropped from subsequent analyses because they had extremely low item-total correlations or high coefficients on more than one factor. The final model contained eight factors, as confirmed by parallel analysis, Velicer MAP, and BIC.

The factor pattern and factor structure coefficients are presented in Table 2, along with communalities (*h*^2^) of the measured variables. All 29 items had communalities of at least 0.60 and above [29]. It should be noted that the pattern coefficient of some factors is slightly greater than 1.0 due to the use of an oblique rotation, which allows the factors to correlate; this does not imply an error but rather indicates a high degree of multicollinearity in the data [30].

**Table 2:** Pattern matrix, structure matrix, and communalities.

	Data Awareness		Data Collection		Data Analytics		Data Visualization		Data Storytelling		Data Quality Evaluation		Data Management		Data Ethics		
Item	P	S	P	S	P	S	P	S	P	S	P	S	P	S	P	S	h^2^
DA1.1	**0.88**	0.84	0.01	0.54	−0.10	0.47	0.01	0.49	0	0.42	0	0.49	0.03	0.41	−0.01	0.33	0.71
DA1.2	**0.9**	0.89	−.006	0.52	0.08	0.59	0.03	0.55	−0.07	0.43	0	0.54	−0.04	0.46	0.04	0.42	0.81
DA1.3	**0.7**	0.81	0.07	0.56	0.07	0.55	−0.02	0.52	0.05	0.48	−0.01	0.53	0	0.45	0.02	0.4	0.66
DCO2.1	0.1	0.51	**0.59**	0.71	−0.07	0.29	0.08	0.46	0.07	0.44	0.01	0.44	−0.02	0.24	0	0.24	0.51
DCO2.2	−0.07	0.52	**1.06**	0.95	−0.03	0.26	0.05	0.46	−0.07	0.41	−0.08	0.43	−0.06	0.19	0.07	0.23	0.92
DCO2.3	−0.03	0.52	**0.71**	0.75	0.13	0.41	−0.01	0.45	−0.07	0.41	0.06	0.51	0.07	0.36	0	0.3	0.59
DCL3.1	0.17	0.64	0.10	0.45	**0.72**	0.83	−0.14	0.51	0.03	0.49	0.08	0.62	0.08	0.61	−0.11	0.45	0.74
DCL3.2	0.01	0.57	0.08	0.38	**0.87**	0.88	−0.14	0.5	0.08	0.5	−0.02	0.59	0.15	0.65	−0.11	0.48	0.81
DCL3.3	−0.07	0.50	0.09	0.32	**1.02**	0.9	−0.24	0.43	0.07	0.46	−0.03	0.55	0.09	0.63	−0.05	0.49	0.85
DA4.2	0.01	0.51	0.00	0.35	**0.67**	0.75	0.32	0.65	−0.03	0.48	−0.04	0.52	−0.17	0.42	0.05	0.47	0.64
DA4.3	−0.02	0.48	−0.06	0.29	**0.75**	0.79	0.29	0.62	−0.07	0.45	−0.03	0.51	−0.16	0.44	0.07	0.49	0.67
DA4.4	−0.05	0.45	−0.13	0.20	**0.81**	0.82	0.13	0.54	−0.05	0.42	0.01	0.52	−0.04	0.53	0.1	0.53	0.70
DV5.1	0.07	0.59	−0.03	0.47	0.05	0.61	**0.75**	0.85	0	0.6	0.01	0.62	0.15	0.5	−0.07	0.44	0.74
DV5.2	−0.03	0.57	0.14	0.57	−0.04	0.43	**0.83**	0.89	0.07	0.65	−0.05	0.61	0.08	0.42	−0.04	0.43	0.81
DV5.3	−0.01	0.57	0.04	0.52	0.05	0.61	**0.65**	0.85	0.1	0.67	0.09	0.67	0.06	0.49	−0.01	0.49	0.75
DS6.1	0	0.53	0.11	0.56	0.04	0.53	0.23	0.72	**0.63**	0.84	0.01	0.63	−0.06	0.39	−0.01	0.46	0.75
DS6.2	−0.02	0.42	−0.10	0.38	0.02	0.5	0.01	0.59	**0.88**	0.87	0.01	0.59	0.04	0.43	0.01	0.48	0.77
DS6.3	−0.01	0.45	−0.02	0.45	−0.01	0.48	−0.03	0.61	**0.98**	0.94	−0.01	0.61	−0.05	0.39	0.06	0.5	0.88
DQE7.1	0.02	0.57	0.01	0.51	0.01	0.6	0.11	0.64	−0.02	0.6	**0.79**	0.87	0.02	0.57	−0.02	0.49	0.77
DQE7.2	−0.01	0.54	−0.06	0.49	−0.06	0.57	−0.03	0.59	0.01	0.63	**1.08**	0.96	−0.02	0.57	−0.02	0.5	0.93
DQE7.3	−0.01	0.56	0.06	0.53	0.07	0.62	−0.04	0.6	0.04	0.63	**0.77**	0.88	−0.02	0.58	0.07	0.55	0.79
DO8.3	0	0.47	−0.07	0.26	0.32	0.69	0.06	0.48	−0.01	0.44	0.03	0.56	**0.41**	0.71	0.12	0.57	0.60
DS9.1	0.06	0.52	0.00	0.32	−0.06	0.61	0.12	0.47	−0.04	0.43	−0.04	0.58	**0.93**	0.92	−0.03	0.53	0.85
DS9.2	−0.05	0.47	0.04	0.32	−0.06	0.6	0.07	0.45	−0.02	0.44	0.01	0.61	**0.95**	0.93	0.02	0.57	0.88
DS9.3	−0.02	0.46	−0.07	0.25	0.09	0.66	0.02	0.44	0.03	0.45	−0.01	0.59	**0.8**	0.9	0.08	0.81	0.82
DE10.1	0.04	0.37	0.07	0.30	−0.13	0.41	0.02	0.42	0.03	0.45	−0.01	0.45	−0.05	0.43	**0.86**	0.81	0.67
DE10.2	0.03	0.40	−0.03	0.24	0.11	0.56	−0.07	0.42	0	0.46	0.01	0.49	−0.03	0.53	**0.87**	0.89	0.79
DE10.3	−0.02	0.39	0.03	0.28	0	0.54	−0.03	0.45	0.03	0.49	−0.02	0.51	0.03	0.56	**0.91**	0.92	0.85
DE10.4	−0.02	0.39	0.00	0.25	0.06	0.57	−0.06	0.42	0	0.46	−0.02	0.52	0.16	0.62	**0.75**	0.85	0.75
Eigenvalue	2.19		2.12		4.44		2.46		2.34		2.42		2.97		3.07		
% of Variance	8%		7%		15%		8%		8%		8%		10%		11%		

Notes: P = pattern coefficients; S = structure coefficients; h^2^ = communalities of the measured variables.

Pattern coefficients with values of .40 or greater are in bold.

For the full statements corresponding to their respective item numbers, please refer to Appendix 1 in the S1 Appendix.

The first factor, *Data Awareness*, has high loadings on three items related to understanding the foundational concepts and use of data. The second factor, *Data Collection*, comprises three items covering topics such as identifying the source of data and using appropriate methods to collect data. The third factor, *Data Analytics*, has six items with factor loadings ranging from 0.67 to 1.02. This factor reflects items on methods and techniques in data cleaning and analysis. The fourth factor, labeled *Data Visualization*, consists of three items that pertain to data visualization methods and the interpretation of visualization. The fifth factor is *Data Storytelling*, which includes three items about presenting and communicating data insights to others using different methods or techniques. The sixth factor, *Data Quality Evaluation*, consists of three items with factor loadings of 0.77, 0.79, and 1.08. The items are centered on the evaluation of data use and data quality. The seventh factor, *Data Management*, has four items relevant to the documentation and storage of data for share and reuse. The item DO 8.3 has a relatively low factor loading of 0.41; this implies that this specific item has a moderate relationship with the broader concept of data management and could indicate that the item is less critical or less directly related to the core components of data management. The last factor identified is *Data Ethics*, which comprises four items focused on understanding laws, regulations, and guidelines relevant to data use in terms of privacy, security, and intellectual property. The items have loadings ranging from 0.75 to 0.91.

A reliability analysis was conducted to assess the scales’ internal consistency. The Cronbach’s alpha ranges from 0.77 to 0.90 for the eight factors, which all exceeded the acceptable threshold of 0.70 [31], indicating good internal consistency.

### Confirmatory factor analysis results

The 29-item model with an eight-factor solution extracted from the EFA was used for the CFA. The model was structured with each item assigned to its corresponding factor, and the factors were allowed to correlate. This is based on the EFA results and reflects the theoretical perspective that the factors are distinct but interrelated. The model’s fit was assessed through the fit indices discussed in the Data Analysis section of this paper, all of which indicated that the model fit the data well; χ^2^ (349) = 1225.60, *p* <.001. The model fit had a good model fit, CFI = 0.994, TLI = 0.994, RMSEA = 0.053, SRMR = 0.044.

Average variance extracted (AVE) ranged from 0.64 to 0.82 across the eight factors, which is above the 0.50 recommendation [21]. This indicated that between 64% and 82% of the indicator variance is part of the construct score. The composite reliabilities with a correction for the ordinal nature of the items were extracted and ranged from 0.8 to 0.95 across the eight factors, above the 0.70 recommendation [32].

**Fig 1:**
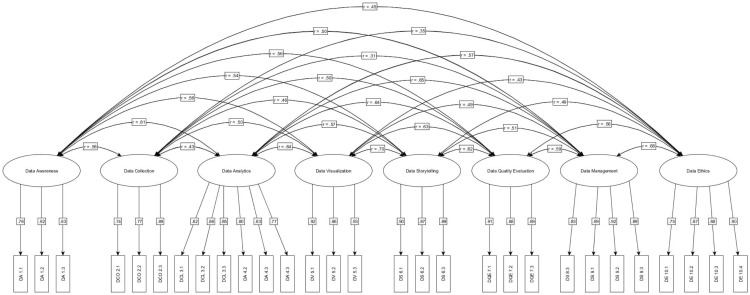
Confirmatory factor analysis model.

Fig 1 denotes the CFA results. The standardized loadings of all items were in the range of 0.74 to 0.92, indicating strong associations. All items’ loadings were statistically significant (*p* < 0.001), which confirms that the items are good indicators of their respective factors. The correlation among the scale factors is between 0.31 and 0.70, and the correlation between each factor is significant (*p* < 0.01). This indicates that eight factors constitute the components of students’ self-assessed data literacy, but they are related constructs.

### Item analysis results

Psychometric analysis at the item level is presented in Table 3, which shows the descriptive statistics for the items, item-total correlations, and the Cronbach’s alpha coefficients. DCO 2.1 “Collecting primary data...” and DCO 2.2 “Locating secondary data...” have the highest mean among all items, whereas DA 4.4 “Applying data mining to explore patterns and trends of data” has the highest standard deviation. Items belonging to *Data Management* and *Data Ethics* exhibit slightly lower means and higher standard deviations than other items.

**Table 3:** Item-level analysis results.

				Likert Response Frequencies N (%)				
Factor	Item	Mean (SD)	Item-total Correlation	1	2	3	4	5
Data Awareness α =.81	DA 1.1	4.17 (.65)	.81	0 (0%)	9 (1%)	102 (11.2%)	521 (57.4%)	276 (30.4%)
	DA 1.2	3.98 (.83)	.89	0 (0%)	59 (6.5%)	146 (16.1%)	456 (50.2%)	247 (27.2%)
	DA 1.3	4.04 (.79)	.87	0 (0%)	46 (5.1%)	130 (14.3%)	476 (52.4%)	256 (28.2%)
Data Collection α =.76	DCO 2.1	4.27 (.69)	.76	0 (0%)	11 (1.2%)	90 (9.9%)	447 (49.2%)	360 (39.6%)
	DCO 2.2	4.21 (.71)	.86	0 (0%)	17 (1.9%)	106 (11.7%)	458 (50.4%)	327 (36%)
	DCO 2.3	4.10 (.81)	.84	0 (0%)	38 *4.2%)	146 (16.1%)	415 (45.7%)	309 (34%)
Data Analytics α =.88	DCL 3.1	3.73 (.92)	.76	5 (0.6%)	109 (12%)	184 (20.3%)	440 (48.5%)	170 (18.7%)
	DCL 3.2	3.65 (.94)	.82	5 (0.6%)	122 (13.4%)	218 (24%)	405 (44.6%)	158 (17.4%)
	DCL 3.3	3.46 (1.02)	.84	11 (1.2%)	175 (19.3%)	257 (28.3%)	318 (35%)	147 (16.2%)
	DA 4.2	3.83 (.91)	.76	6 (0.7%)	84 (9.3%)	174 (19.2%)	435 (47.9%)	209 (23%)
	DA 4.3	3.69 (.97)	.78	9 (1%)	118 (13%)	208 (22.9%)	387 (42.6%)	186 (20.5%)
	DA 4.4	3.40 (1.13)	.79	31 (3.4%)	216 (23.8%)	186 (20.5%)	313 (34.5%)	162 (17.8%)
Data Visualization α =.87	DV 5.1	4.06 (.77)	.89	0 (0%)	44 (4.8%)	116 (12.8%)	493 (54.3%)	255 (21.8%)
	DV 5.2	4.17 (.73)	.89	0 (0%)	24 (2.6%)	103 (11.3%)	475 (52.3%)	306 (33.7%)
	DV 5.3	4.09 (.74)	.90	0 (0%)	31 (3.4%)	120 (13.2%)	492 (54.2%)	265 (29.2%)
Data Storytelling α =.86	DS 6.1	4.05 (.75)	.84	0 (0%)	30 (3.3%)	140 (15.4%)	491 (54.1%)	247 (27.2%)
	DS 6.2	3.81 (.93)	.90	5 (0.6%)	90 (9.9%)	195 (21.5%)	405 (44/6%)	213 (23.5%)
	DS 6.3	3.93 (.86)	.91	3 (0.3%)	61 (6.7%)	173 (19.1%)	434 (47.8%)	237 (26.1%)
Data Quality Evaluation α =.87	DQE 7.1	3.93 (.78)	.89	0 (0%)	48 (5.3%)	169 (18.6%)	493 (54.3%)	198 (21.8%)
	DQE 7.2	3.94 (.78)	.90	0 (0%)	50 (5.5%)	156 (17.2%)	500 (55.1%)	202 (22.2%)
	DQE 7.3	3.87 (.80)	.89	0 (0%)	53 (5.8%)	196 (21.6%)	476 (52.4%)	183 (20.2%)
Data Management α =.89	DO 8.3	3.65 (1.09)	.78	24 (2.6%)	139 (15.3%)	189 (20.8%)	333 (36.7%)	223 (24.6%)
	DS 9.1	3.76 (.99)	.87	12 (1.3%)	106 (11.7%)	192 (21.1%)	374 (41.2%)	224 (24.7%)
	DS 9.2	3.62 (1.04)	.90	17 (1.9%)	140 (15.4%)	205 (22.6%)	359 (39.5%)	187 (20.6%)
	DS 9.3	3.49 (1.09)	.90	32 (3.5%)	152 (16.7%)	237 (26.1%)	315 (34.7%)	172 (18.9%)
Data Ethics α =.89	DE 10.1	3.71 (1.01)	.81	25 (2.8%)	96 (10.6%)	194 (21.4%)	394 (43.4%)	199 (21.9%)
	DE 10.2	3.44 (1.12)	.88	39 (4.3%)	179 (19.7%)	193 (21.3%)	334 (36.8%)	163 (18%)
	DE 10.3	3.52 (1.09)	.88	35 (3.9%)	150 (16.5%)	205 (22.6%)	343 (37.8%)	175 (19.3%)
	DE 10.4	3.41 (1.14)	.88	44 (4.8%)	180 (19.8%)	214 (23.6%)	302 (33.3%)	168 (18.5%)

All items had item-total correlations of at least 0.76, well above the acceptable threshold of 0.40 [33]; this indicates that each item demonstrated satisfactory discrimination. The coefficient value for each factor ranged from 0.76 to 0.89, exceeding the acceptable Cronbach’s alpha level of 0.70 for scale development; this indicates strong convergence and internal consistency.

## Discussion

This study was the first to examine the factor structure of the DLSES among students enrolled at four-year institutions. Given the variability of factor structure across different populations [23], EFA was used as an inductive process to identify a factor structure that may be unique to university students. Indeed, the study resulted in eight factors on the scale, which were significantly correlated with one another: *Data Awareness*, *Data Collection*, *Data Analytics*, *Data Visualization*, *Data Storytelling*, *Data Quality Evaluation*, *Data Management*, and *Data Ethics*. A subsequent CFA supported the factors found in the EFA; this proves that the DLSES measured and reflected the aforementioned eight latent components, providing empirical evidence to build the construct validity of the scale.

The factor structure derived from the current study differed from the previous study done with community college students [14]. This difference could arise from differences in both exposure and experience with data literacy and actual competency levels among various populations. Due to their longer and more specialized academic training, students at four-year institutions may have a higher level of proficiency and familiarity with data-related concepts and skills. Therefore, they may show a more refined and comprehensive understanding of data literacy than community college students. This is further supported by the results showing that the mean scores of items across the scale for university students range from 3.40 to 4.27, higher than the scores of community college students in the previous study, which ranged from 2.39 to 2.99 [14].

It is also important to highlight that the *Data Management* and *Data Ethics* competencies have lower mean scores compared to other areas. This is likely because these competencies are often less emphasized in data literacy. Additionally, the fact that 69% of the sample consists of students in STEM fields, who typically engage more directly with data than non-STEM students, may also contribute to this result. Their coursework and research frequently involve data analysis, interpretation, and the application of statistical methods, leading to stronger data skills due to the emphasis on quantitative analysis and problem-solving in STEM disciplines. The slightly higher standard deviations observed for *Data Management* and *Data Ethics* also reflect variability in participants’ responses, which may be attributed to differences in understanding, experiences, or beliefs regarding these competencies. This suggests that more attention should be given to developing these competencies, which often require critical thinking and a deeper understanding of how to manage data effectively and handle it responsibly. This approach will help bridge the observed gaps and improve overall data literacy in education.

### Limitations and future recommendations

A limitation of this study is the potential for biased sample selection, which may affect the reliability of the findings. Given that participation in the survey was self-selected, it is possible that those who volunteered had a specific interest in data literacy or had confidence in their data skills. To address this, further psychometric evaluations are needed to validate the scale across different groups. Future research should include a more diverse and representative sample from various academic disciplines and consider cross-cultural validation to improve generalizability.

Moreover, it is worth emphasizing that scale development and validation are iterative processes involving multiple stages of refinement and testing to ensure reliability and validity [33]. Future studies should seek feedback from experts and potential users to improve the scale’s clarity, relevance, and comprehensiveness. Additionally, updates to data literacy competencies may be needed as the field evolves.

## Conclusions

The DLSES validated in this study is anticipated to become a promising tool for researchers and educators. Researchers can use the scale to gauge individuals’ confidence in their skills to understand, analyze, and use data effectively across different academic disciplines. Also, given that there is a call for more proactive approaches to integrating data literacy training into current higher education, the scale can be used to screen students’ data literacy competency as a preliminary needs assessment. Educators can use this scale to identify areas where students may need additional support or instruction, ultimately helping students become more proficient and confident in navigating data.

Data literacy education is an emerging field of study, and there is a need for more strategies and best practices in teaching data literacy. This involves equipping students with the technical skills required to analyze and interpret data and fostering a deeper understanding of the context in which data is used. Moreover, given the growing emphasis on “critical data literacy” [34,35], the scope of data literacy education must expand beyond questioning data sources, analytical methods, and conclusions. It should also include understanding the origins of data, the perspectives of those managing it, the broader implications of data analysis and interpretation, and the ethical dimensions of its use.

## Supporting information

S1 Appendix Scale items. (DOCX)
