# Supplementary material for: University students’ self-assessment of data literacy: A validation study
Source: PLoS One. 2025 Apr 28;20(4):e0322104. doi: 10.1371/journal.pone.0322104 (PMC12036854; doi:10.1371/journal.pone.0322104)
Supplement: S1 Appendix — (DOCX) [file pone.0322104.s001.docx]

Appendix 1. Scale items

| Item | Statement |
| --- | --- |
| DA 1.1 | Define what data is |
| DA 1.2 | Describe different types and formats of data |
| DA 1.3 | Explain the real and potential value of data |
| DCO 2.1 | Collect primary data, such as surveys, observations, experiments, questionnaires, or interviews |
| DCO 2.2 | Locate secondary data from print/electronic sources, such as journal articles and government publications |
| DCO 2.3 | Access/locate secondary data from electronically stored information, such as websites, APIs, and databases |
| DCL 3.1 | Identify data errors, including corrupted, incorrectly formatted, duplicate, or incomplete data |
| DCL 3.2 | Correct data errors using appropriate tools and techniques |
| DCL 3.3 | Apply data integration techniques to heterogeneous and distributed data |
| DA4.1 | Display/summarize data using descriptive data |
| DA 4.2 | Use inferential statistics to draw meaningful inferences from data |
| DA 4.3 | Employ predictive analytics to make predictions and direct decisions |
| DA 4.4 | Apply data mining to explore patterns and trends of data |
| DA 4.5 | Communicate in writing and verbally the results of data analysis/analytics |
| DV 5.1 | Select appropriate tools to create data visualizations |
| DV 5.2 | Create useful and meaningful data visualizations using charts, tables, and graphics using a chosen dataset |
| DV 5.3 | Integrate data visualizations to highlight key points of interest |
| DS 6.1 | Present data insights tailored to a specific audience |
| DS 6.2 | Use a storyboarding technique to plan the content and structure of a data presentation |
| DS 6.3 | Use a narrative structure to create compelling data presentations |
| DQE 7.1 | Assess data sources to ensure they meet the defined need |
| DQE 7.2 | Determine whether data meets the quality required for a given purpose |
| DQE 7.3 | Evaluate data critically for the right type and quantity to support its intended use |
| DO 8.1 | Categorize/classify data to make it more accessible for use |
| DO8.2 | Organize/distribute data among files, including file naming conventions, directory structure, version control, etc. |
| DO 8.3 | Document data to improve data findability and accessibility (e.g., codebook, data dictionary, README file, metadata) |
| DS 9.1 | Differentiate between different data storage devices |
| DS 9.2 | Identify various data backup methods and strategies |
| DS 9.3 | Determine the best storage options for short- and long-term data storage |
| DE 10.1 | Locate laws, regulations, and guidelines, such as FERPA, HIPAA, and GDPR, for handling data security and privacy |
| DE 10.2 | Utilize de-identification techniques to protect personal data |
| DE 10.3 | Determine data ownership and use rights – i.e., intellectual property rights and licensing |
| DE 10.4 | Identify current best practices in data citation, including the use of permanent identifiers, notably Digital Object Identifiers |
